# Supplementary material for: The Effect of Increasing Levels of Dehulled Faba Beans (Vicia faba L.) on Extrusion and Product Parameters for Dry Expanded Dog Food
Source: Foods. 2019 Jan 12;8(1):26. doi: 10.3390/foods8010026 (PMC6351981; doi:10.3390/foods8010026)
Supplement: Supplementary file 1 [file foods-08-00026-s001.pdf]

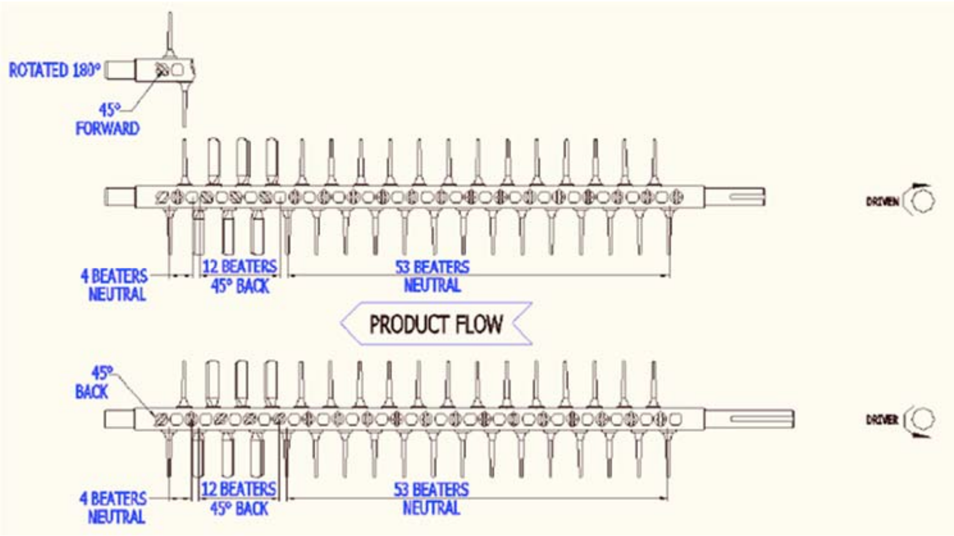

Figure S1. 16x72 DDC preconditioner configuration used to produce FB0, FB10, FB20 and FB30 diets.

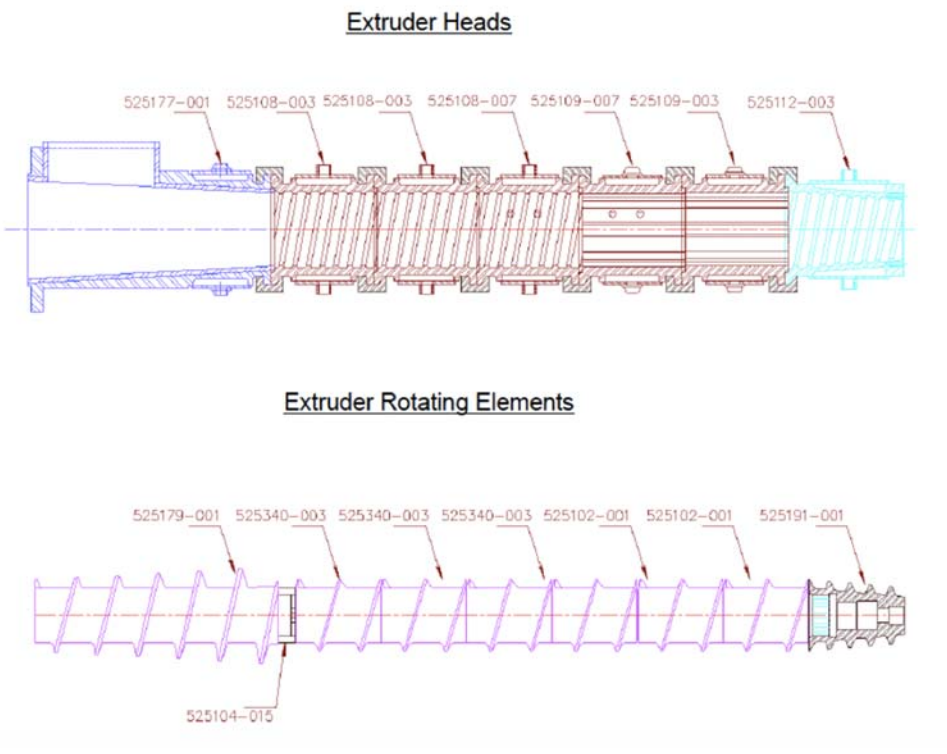

Figure S2. Extruder barrel configuration used to produce experimental diets.
